# Cyclic di-GMP Signaling in Bacillus subtilis Is Governed by Direct Interactions of Diguanylate Cyclases and Cognate Receptors

**DOI:** 10.1128/mBio.03122-19

**Published:** 2020-03-10

**Authors:** Sandra Kunz, Anke Tribensky, Wieland Steinchen, Luis Oviedo-Bocanegra, Patricia Bedrunka, Peter L. Graumann

**Affiliations:** a SYNMIKRO, LOEWE Center for Synthetic Microbiology, Marburg, Germany; b Department of Chemistry, Philipps-Universität Marburg, Marburg, Germany; National Institute of Child Health and Human Development (NICHD)

**Keywords:** cyclic-di-GMP signaling, biofilm formation, *Bacillus subtilis*, single-molecule dynamics, second messenger, signal transduction, single-molecule tracking

## Abstract

Bacillus subtilis contains two known cyclic di-GMP (c-di-GMP)-dependent receptors, YdaK and DgrA, as well as three diguanylate cyclases (DGCs): soluble DgcP and membrane-integral DgcK and DgcW. DgrA regulates motility, while YdaK is responsible for the formation of a putative exopolysaccharide, dependent on the activity of DgcK. Using single-molecule tracking, we show that a majority of DgcK molecules are statically positioned in the cell membrane but significantly less so in the absence of YdaK but more so upon overproduction of YdaK. The soluble domains of DgcK and of YdaK show a direct interaction *in vitro*, which depends on an intact I-site within the degenerated GGDEF domain of YdaK. These experiments suggest a direct handover of a second messenger at a single subcellular site. Interestingly, all three DGC proteins contribute toward downregulation of motility via the PilZ protein DgrA. Deletion of *dgrA* also affects the mobility of DgcK within the membrane and also that of DgcP, which arrests less often at the membrane in the absence of DgrA. Both, DgcK and DgcP interact with DgrA *in vitro*, showing that divergent as well as convergent direct connections exist between cyclases and their effector proteins. Automated determination of molecule numbers in live cells revealed that DgcK and DgcP are present at very low copy numbers of 6 or 25 per cell, respectively, such that for DgcK, a part of the cell population does not contain any DgcK molecule, rendering signaling via c-di-GMP extremely efficient.

## Abstract (precis)

Second messengers are free to diffuse through the cells and to activate all responsive elements. Cyclic di-GMP (c-di-GMP) signaling plays an important role in the determination of the life style transition between motility and sessility/biofilm formation but involves numerous distinct synthetases (diguanylate cyclases [DGCs]) or receptor pathways that appear to act in an independent manner. Using Bacillus subtilis as a model organism, we show that for two c-di-GMP pathways, DGCs and receptor molecules operate via direct interactions, where a synthesized dinucleotide appears to be directly used for the protein-protein interaction. We show that very few DGC molecules exist within cells; in the case of exopolysaccharide (EPS) formation via membrane protein DgcK, the DGC molecules act at a single site, setting up a single signaling pool within the cell membrane. Using single-molecule tracking, we show that the soluble DGC DgcP arrests at the cell membrane, interacting with its receptor, DgrA, which slows down motility. DgrA also directly binds to DgcK, showing that divergent as well as convergent modules exist in B. subtilis. Thus, local-pool signal transduction operates extremely efficiently and specifically.

## INTRODUCTION

The bacterial second-messenger cyclic di-GMP (c-di-GMP) plays a key role in the control of bacterial motility and biofilm formation. In general, an increase in intracellular c-di-GMP production correlates with a sessile lifestyle, whereas low c-di-GMP levels favor a planktonic lifestyle. Intracellular c-di-GMP levels are controlled by the antagonistic activity of c-di-GMP-specific synthetases (diguanylate cyclases [DGCs]) and hydrolases (phosphodiesterases [PDEs]) (1). Bacteria contain diverse c-di-GMP binding receptors/effectors that respond to the regulatory functions of this signaling molecule (2, 3).

A given bacterial genome typically contains several genes encoding DGCs and PDEs, which are often involved in very specific, nonoverlapping signal transduction pathways. This leads to the question of how cells cope with such a multiplicity of signaling components and, in parallel, how they guarantee specificity within different signaling modules. Two general models for signal specificity through functional sequestration have been considered: the so-called local- and global-pool signaling hypotheses (4). In Bacillus subtilis, only PdeH has been shown to confer PDE activity *in vitro* and *in vivo*, and it contains no other EAL domain-containing protein, so PdeH appears to control intracellular c-di-GMP levels (5), which argues for a global-pool mechanism. However, many distinct phenotypes of deletions in individual pathways observed for several species cannot easily be explained by a global pool. Instead, spatially sequestering the signal (local pool) within multiprotein complexes at distinct cellular sites may result in highly specific signaling pathways. Evidence for this idea has come from the identification (i) of direct interactions of a DGC and its receptor protein in the Gram-negative organism Pseudomonas fluorescens (6); (ii) of a module of interacting DGCs, PDEs, and a DNA-binding protein acting as a signaling cascade in Escherichia coli (7); and (iii) of single, distinct localization points of DGCs and of effector proteins in B. subtilis (8, 9). A third scenario has recently been suggested based on the identification of interaction hubs of DGCs and PDEs in E. coli, where a few DGCs and PDEs show multiple functional interactions in a tightly interconnected network (10, 11). Temporal and/or conditional separation through differential expression and activation of DGCs, PDEs, and output systems may therefore have a distinct impact on the global c-di-GMP pool and generate network specificity.

**FIG 1:**
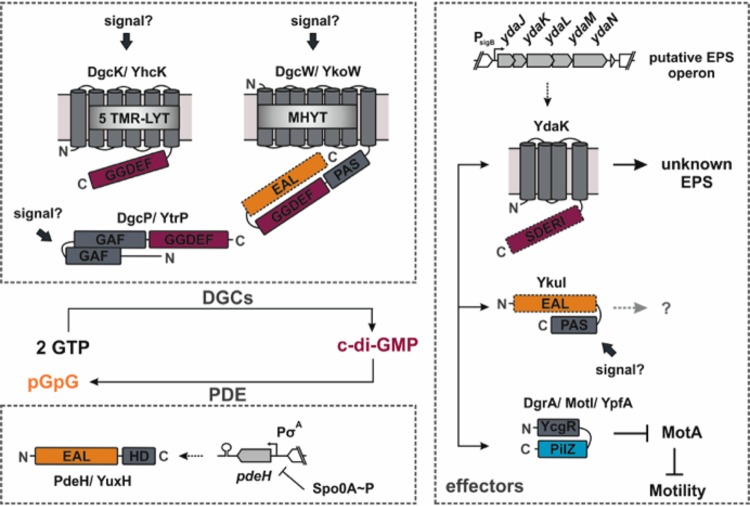
Known components of the c-di-GMP signaling network in B. subtilis. The color code for protein domains is as follows: gray for the TM and putative soluble signaling domains, purple for the GGDEF domains, orange for the EAL domains, and blue for the PilZ domain. Inactive protein domains are illustrated with dashed lines. Note that the EAL domain activity of DgcW has not been tested so far *in vitro*.

The Gram-positive model organism B. subtilis possesses a relatively concise c-di-GMP signaling set (Fig. 1). Three DGCs, DgcK, DgcP, and DgcW, as well as one active PDE, PdeH, control at least two known c-di-GMP signaling pathways (5, 12). One pathway regulates the production of an unknown exopolysaccharide (EPS) (9), and the other adapts motility (5). The gene encoding the c-di-GMP receptor YdaK resides in the putative EPS synthesis operon *ydaJKLMN*. Artificial induction of the *yda* operon results in a strongly altered structure of colony biofilms and cell clumping (9). The putative EPS synthase components YdaM/YdaN and YdaK colocalize to clusters predominantly at the cell poles and are statically positioned at this subcellular site, suggesting that exopolysaccharide production takes place at distinct sites of the membrane. For this activity, the presence of YdaK and of DgcK is required, implying an involvement of the second messenger, c-di-GMP. The cytoplasmic DgcP synthetase can complement DgcK only upon overproduction, while the third c-di-GMP synthetase, DgcW, does not seem to be part of the signaling pathway (9).

A further component of the c-di-GMP network in B. subtilis, the receptor DgrA, operates in a clutch-like mechanism to downregulate motility by binding to MotA and dislodging it from its motor activity at the flagellum (5, 13). Deletion of all DGC-encoding genes leads to the loss of DgrA activity (5). It has been unclear which of the DGCs provides the signal for DgrA.

Based on the spatial proximity of DgcK and YdaK (9), we set out to gather additional information on whether the local-pool signaling hypothesis may be operative. Because interactions of membrane proteins are inherently difficult to study, we gathered information of the movement of DgcK within the cell membrane, using single-molecule tracking, which allows one to determine average times spent in a bound (static/slow moving) or unbound (freely diffusive) mode, as well as the spatial preference for movement within subcellular sites and single-cell protein copy numbers. The rationale is that the movement of DgcK should be affected by the presence or absence of a binding partner, in this case of YdaK. Furthermore, we investigated which of the DGCs might mediate inhibition of motility via the c-di-GMP receptor DgrA and whether, also in this case, protein dynamics are affected in the absence of DgrA, which we found to be the case. Our data of pulldown and hydrogen-deuterium exchange mass spectrometry (HDX-MS) experiments using purified soluble parts of DGCs and receptors support the notion of c-di-GMP signaling involving convergent and divergent direct protein-protein interactions.

## RESULTS

### The fraction of static DgcK molecules and their dwell times depends on the availability of YdaK.

Membrane-integral cyclase DgcK has been shown to form clusters in the cell membrane, which can colocalize with the single YdaK/YdaNM cluster, indicating the close spatial proximity of c-di-GMP synthetase and its receptor (8). We wished to strengthen the view that a close interaction exists between DgcK and YdaK by determining single-molecule dynamics of DgcK in the presence or absence of YdaK. In case YdaK serves as a direct or indirect binding partner, DgcK movement should increase in the absence of YdaK.

**FIG 2:**
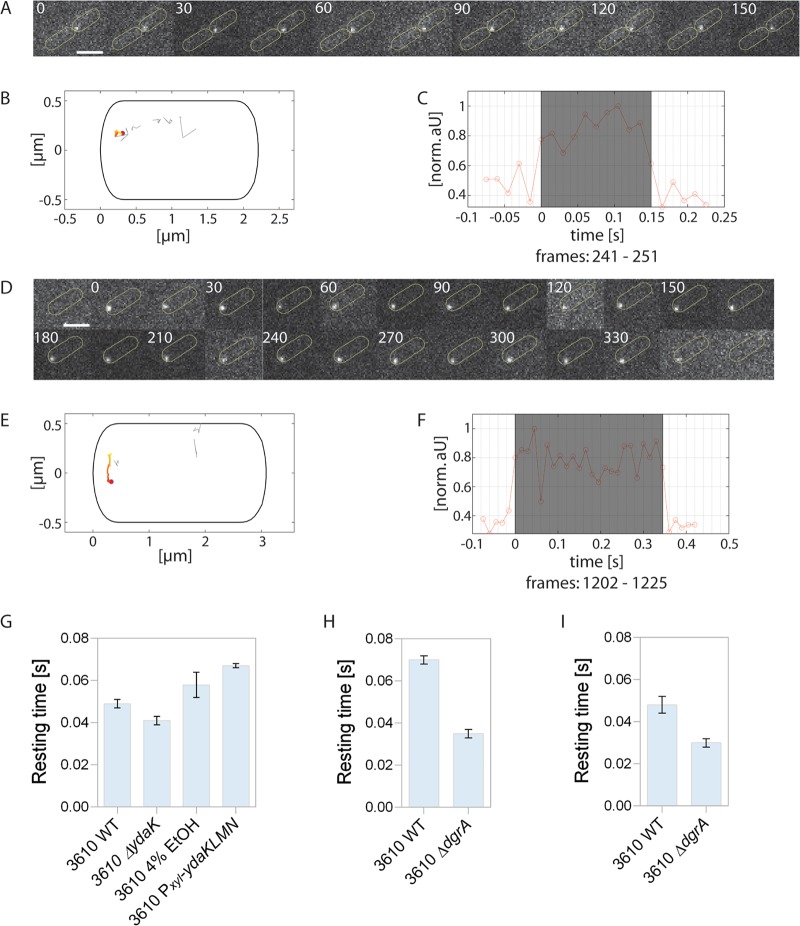
Single-molecule tracking of exponentially growing DgcK-mVenus in B. subtilis 3610 wild-type cells. (A) Representative static molecule of DgcK-mVenus in the wild type localized at the cell pole (D), and dynamic track of DgcK-mVenus moving along the cell pole. Scale bars correspond to 2 μm. (B, E) Corresponding projections of all tracks in these cells showing the static (B) or mobile (E) track in a color-coded manner. The origin is highlighted in red and the end in yellow. All other tracks in the same cell are displayed in gray. (C, F) Normalized intensity profile of the track shaded in gray. (G to I) Average dwell times of DgcK-mVenus and DgcP-mVenus in different B. subtilis NCIB 3610 strains. (G) The bar plots depict the change in the average dwell times (calculated with “SMM Track”) of DgcK-mVenus in the NCIB 3610 wild type (3610 WT), in a *ydaK* deletion strain (3610 Δ*ydaK*), and in two strains overexpressing *ydaK* (3610 4% EtOH, cells stressed with 4% ethanol for 30 min; 3610 P*_xyl_*-*ydaKLMN*, NCIB 3610 with P*_xyl_*-*ydaKLMN*). The absence of YdaK leads to a decrease of the dwell time, whereas overexpression leads to an increase. (H, I) Dwell times of DgcK-mVenus (H) or DgcP-mVenus (I) in a stain lacking the c-di-GMP receptor *dgrA* (3610 Δ*dgrA*) and in the NCIB 3610 wild type. All data are from three biological replicates.

A DgcK-mVenus fusion was shown to be functional (8) and was tracked at a 15-ms stream acquisition (see Movie S1 in the supplemental material). Figure 2 shows the two general forms of movement for DgcK-mVenus: free diffusion within the membrane and static motion. Figure 2A displays a sequence of a single molecule that shows very little displacement (gray tracks indicate molecules with more displacement [Fig. 2B]), while Fig. 2D and E show a mobile molecule that moves along the cell pole. Both molecules start fluorescing between two frames and bleach within a single frame (Fig. 2C and F). Note that in order to track membrane protein DgcK, the focal plane was above the center of the cell, so tracks can move seemingly within the cell.

10.1128/mBio.03122-19.8 MOVIE S1 Exponentially growing B. subtilis cells expressing DgcK-mVenus from the original gene locus. Stream acquisition at 15 ms (66 frames per second [fps]) is shown in slow motion at 10 fps. Download Movie S1, AVI file, 0.8 MB. Copyright © 2020 Kunz et al. 2020 Kunz et al. This content is distributed under the terms of the Creative Commons Attribution 4.0 International license.

**FIG 3:**
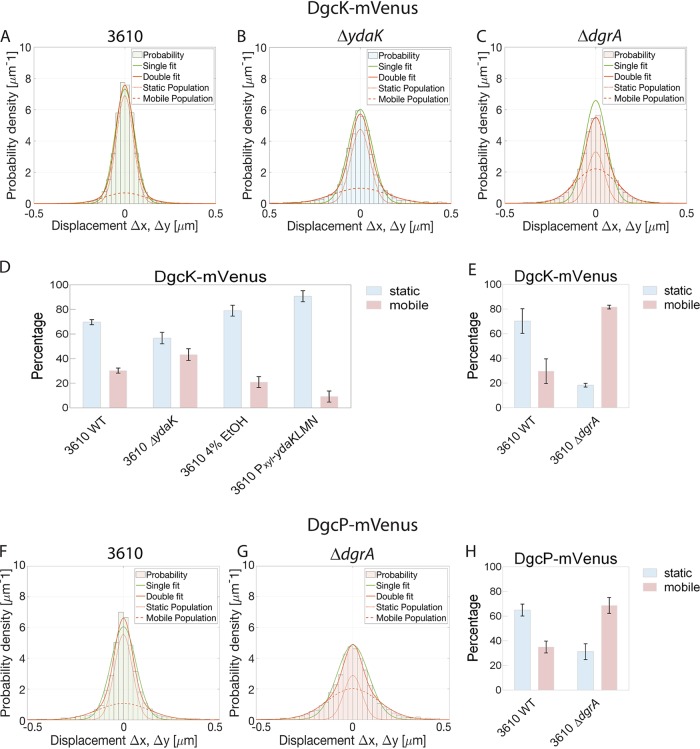
Analyses of the mobility of DGC DgcK and DgcP by the Gaussian mixture model (GMM) in different B. subtilis NCIB 3610 strains. For the determination of fraction sizes and of diffusion coefficients, the GMM was used. (A to C) GMM of DgcK-mVenus in NCIB 3610 (wild-type) (A), NCIB 3610 Δ*ydaK* (B), and NCIB 3610 Δ*dgrA* (C) cells. The histogram of the step-size distribution was fitted with a single fit and a double fit, which consists of a static/slow population and a mobile population, as indicated in the key. Because the double fit corresponds better to the data than the single fit, the double fit was taken for all determinations. (D, E) The bar plots depict the changes in the distributions of the two (static and mobile) subpopulations of DgcK-mVenus. (D) In the absence of the c-di-GMP receptor YdaK (NCIB 3610 Δ*ydaK*), the size of the static DgcK-mVenus population decreases, whereas this population increased upon overexpression of YdaK (NCBI 3610 cells stressed with 4% ethanol for 30 min, or NCIB3610 Pxyl-ydaKLMN cells overexpressing YdaK via addition of xylose). (E) Deletion of the gene encoding the PilZ domain protein DgrA (NCIB 3610 Δ*dgrA*) leads to an increase of the mobile population of DgcK-mVenus compared to that of the wild type. Data are analyzed from three biological replicates. (F, G) GMM of DgcP-mVenus in NCIB 3610 (F) and NCIB 3610 Δ*dgrA* (G). (H) The bar plots depict the changes in the two subpopulations of DgcP-mVenus. Deletion of the *dgrA* gene encoding the c-di-GMP receptor DgrA (strain 3610 Δ*dgrA*) leads to an increase in the size of the mobile population of DgcK-mVenus compared to that of the wild type.

Figure 3 shows the analyses of the distribution of step sizes determined for DgcK-mVenus in wild-type and mutant cells. The histogram is analyzed according to the Gaussian mixture model, where either a single, double, or triple Gaussian distribution fits the obtained data. It is clear to see that a single fit (green line) cannot account for all data, while the double fit (red line) satisfactorily captures the obtained results, assuming static and mobile molecules (Fig. 3A to C). Accordingly, a majority (70% ± 2%) of DgcK-mVenus molecules are statically positioned in the membrane, with a diffusion coefficient of 0.079 ± 0.001 μm^2^/s, while 30% ± 2% of molecules are mobile within the membrane and move at a coefficient of 0.657 ± 0.081 μm^2^/s (Table 1). Note that these determined coefficients must be multiplied by a factor of 1.23 to correct for the curvature of the membrane (14). The distribution of DgcK is strongly altered in the absence of YdaK: the steps become much wider, indicating an increased mobility of molecules (Fig. 3B). Accordingly, more DgcK molecules become mobile (43% ± 5%) in the absence of YdaK, and fewer molecules are static (57% ± 5%) indicating a loss of binding sites for DgcK (Table 1). YdaK, together with YdaN and YdaM, a putative EPS machinery, forms static clusters in the cell membrane (8). Binding of DgcK to these immobile clusters renders DgcK immobile, which is observed in our imaging.

**TABLE 1:** Determined diffusion coefficients and dwell times of DgcK-mVenus in different B. subtilis NCIB 3610 strains^*a*^

Strain with DgcK-mVenus	Static *D* ± SD (μm² sˉ¹)	Mobile *D* ± SD (μm² sˉ¹)	Static fraction ± SD (%)	Mobile fraction ± SD (%)	Dwell time (s)
NCIB 3610^*b*^	0.079 ± 0.001	0.657 ± 0.081	69.7 ± 2.1	30.3 ± 2.1	0.049 ± 0.002
Δ*ydaK* strain	0.079 ± 0.001	0.657 ± 0.081	56.7 ± 4.7	43.3 ± 4.7	0.041 ± 0.002
Stressed with 4% EtOH	0.079 ± 0.001	0.657 ± 0.081	79.0 ± 4.4	21.0 ± 4.4	0.058 ± 0.006
P*_xyl_-ydaKLMN* strain	0.079 ± 0.001	0.657 ± 0.081	90.7 ± 4.5	9.3 ± 4.5	0.067 ± 0.001
NCIB 3610^*c*^	0.075 ± 0.004	0.383 ± 0.040	70.3 ± 10.0	29.7 ± 10.0	0.070 ± 0.002
Δ*dgrA* strain	0.075 ± 0.004	0.383 ± 0.040	18.3 ± 1.5	81.7 ± 1.5	0.035 ± 0.002

a *D*, diffusion coefficient; dwell time, time period in which a track stays within a radius of 230 nm (2.3 pixels). The errors were calculated from the results of three biological replicates.

b Wild type for *ydaK* experiments.

c Wild type for *dgrA* experiments.

We also determined dwell times of DgcK, using the program “SMM Track” (15). As shown in Table 1 and Fig. 2G, average dwell times of static DgcK molecules decreased from 49 ± 2 ms to 41 ± 2 ms in the absence of YdaK. This is in agreement with the idea that 13% of DgcK molecules no longer find a binding partner in the absence of YdaK. It also shows that interactions are very rapid, in the lower millisecond range, although it has to be kept in mind that the true *in vivo* dwell times will be longer as our assay is based on molecules bleaching, which underestimates actual dwell times.

To strengthen these findings, we sought to increase YdaK levels by (i) inducing transcription of the *yda* operon through stress induction (ethanol, SubtiWiki) (16) or (ii) artificial induction via an engineered xylose promoter upstream of *ydaK*. As can be seen from Fig. 3D and Table 1, ethanol stress led to an increase in the static fraction of DgcK, from 70% ± 2% up to 79% ± 4% (note that the number of YdaK molecules increases about 20% under this condition [see below]), as did the addition of 0.1% xylose (the induction level of this promoter is high), which resulted in almost 91% ± 5% static molecules. Average dwell times of DgcK increased from 49 ± 2 ms to 58 ± 6 ms after ethanol stress and to 67 ± 1 ms after full transcriptional induction (Fig. 2G; Table 1). Therefore, intracellular levels of YdaK strongly determine how long DgcK molecules remain statically positioned in the membrane or move through the membrane, corroborating with the analyses of static/dynamic fractions of DgcK.

In order to rule out that general diffusion effects underlie changes in the dynamics of DgcK, we tracked a protein unrelated to c-di-GMP signaling, CdaA, which is involved in cyclic-di-AMP signaling (17). We did not find any considerable change, either in mobility or in localization, in CdaA in the absence of YdaK or of DgrA (Fig. S2; Table 2), further supporting the specificity of the DgcK-YdaK interaction *in vivo*.

**TABLE 2:** Determined diffusion coefficients and dwell times of CdaA-mVenus in different B. subtilis 3610 strains^*a*^

Strain with CdaA-mVenus	Static *D* ± SD (μm² sˉ¹)	Mobile *D* ± SD (μm² sˉ¹)	Static fraction ± SD (%)	Mobile fraction ± SD (%)
NCIB 3610^*b*^	0.048 ± 0.00021	0.680 ± 0.0043	55.00 ± 0.17	45.00 ± 0.17
Δ*ydaK* strain	0.048 ± 0.00021	0.680 ± 0.0043	57.0 ± 0.17	43.0 ± 0.17
NCIB 3610^*c*^	0.040 ± 0.00017	0.370 ± 0.003	59.00 ± 0.23	41.00 ± 0.23
Δ*dgrA* strain	0.040 ± 0.00017	0.370 ± 0.003	61.0 ± 0.23	39.0 ± 0.23

a The errors were calculated from the results of three biological replicates.

b Wild type for *ydaK* experiments.

c Wild type for *dgrA* experiments.

### The absence of YdaK changes the spatial pattern of DgcK.

To employ a third method to analyze DgcK dynamics dependent on the presence or absence of YdaK, we generated heat maps illustrating preferred locations of single molecules in a standardized cell. To this end, all tracks were projected into a 3- by 1-μm-large cell, and patterns of high or low occupancy of subcellular sites are shown by a color gradient. Figure S1 shows that DgcK is mostly localized in the cell membrane as expected but has a preference for certain positions within the cell membrane, predominantly at the poles and the septum (Fig. S1A). Note that due to small changes in the focal plane during acquisition, molecules can also be seen to traverse in a perpendicular manner relative to the long axis of the cell. When *ydaK* is overexpressed artificially or due to ethanol stress, the localization preference becomes even more pronounced at the cell pole; this can best be seen when heat maps are equalized between all data sets (Fig. S1E to H). In the absence of YdaK, DgcK clusters do not markedly change in their pattern (Fig. S1B), in spite of the loss of statically positioned DgcK molecules. Taken together, these data suggest the direct or close interaction of YdaK and DgcK at the cell membrane, favoring the idea of a direct handover of c-di-GMP between cyclase and the receptor.

10.1128/mBio.03122-19.1 FIG S1 Heat maps of DGCs in B. subtilis wild-type cells and mutant strains. All tracks of each strain are projected into a 3- by 1-μm-large cell to visualize the subcellular distribution of the molecules using the SMTracker. The probability that a protein is located at a certain position in the cell is shown in a color-coded manner; the more likely a molecule is present at a specific point, the darker that point. (A to D) Heat maps of DgcK-mVenus in wild-type B. subtilis NCIB 3610 (A), NCIB 3610 Δ*ydaK* (B), NCIB 3610 P*_xyl_*-*ydaKLMN* (C), and wild-type NCIB 3610 stressed with 4% ethanol (induced *ydaK* expression) (D). When overexpressing the c-di-GMP receptor YdaK, the tracks are predominantly located at several positions at the cell membrane compared to where they occur in the *ydaK* deletion strain. (E to H) Same as panels A to D, but all tracks have an apparent diffusion lower than 0.5 μm^2^/s and an *R*^2^ of >0.7. Intensities are equalized between all four data sets. (I, J) Heat maps of DgcP-mVenus with wild-type NCIB 3610 (E) and NCIB 3610 Δ*dgrA* (F). (K, L) Same as panels I and J, but all tracks have an apparent diffusion lower than 0.5 μm^2^/s and an *R*^2^ of >0.7, as well as equalized data sets. Download FIG S1, TIF file, 0.4 MB. Copyright © 2020 Kunz et al. 2020 Kunz et al. This content is distributed under the terms of the Creative Commons Attribution 4.0 International license.

10.1128/mBio.03122-19.2 FIG S2 Single-molecule dynamics of CdaA-mVenus in different B. subtilis 3610 strains. (A to C) GMM of CdaA-mVenus in NCIB 3610 (wild-type) (A), NCIB 3610 Δ*ydaK* (B), and NCIB 3610 Δ*dgrA* (C) cells. The histogram of the step size distribution was fitted with a single fit, shown in green, and a double fit, shown in red, which consists of a static/slow population (dotted red line) and a mobile population (dashed red line). (D, E) Bar plots depict the change in the distributions of the two subpopulations of CdaA-mVenus (static population in blue and mobile population in red). There was no change in population size in the absence of the c-di-GMP receptor YdaK (NCIB 3610 Δ*ydaK*) (D) or DgrA (NCIB 3610 Δ*dgrA*) (E). (F, G) Equalized heat maps of CdaA-mVenus in wild-type B. subtilis NCIB 3610 (A) and NCIB 3610 Δ*ydaK* (B). (H, I) Equalized heat maps of CdaA-mVenus in wild-type B. subtilis NCIB 3610 (A) and NCIB 3610 Δ*dgrA* (B). Download FIG S2, TIF file, 1.1 MB. Copyright © 2020 Kunz et al. 2020 Kunz et al. This content is distributed under the terms of the Creative Commons Attribution 4.0 International license.

### Direct interaction of the purified soluble domains of DgcK and of YdaK depends on an intact I-site within YdaK.

**FIG 4:**
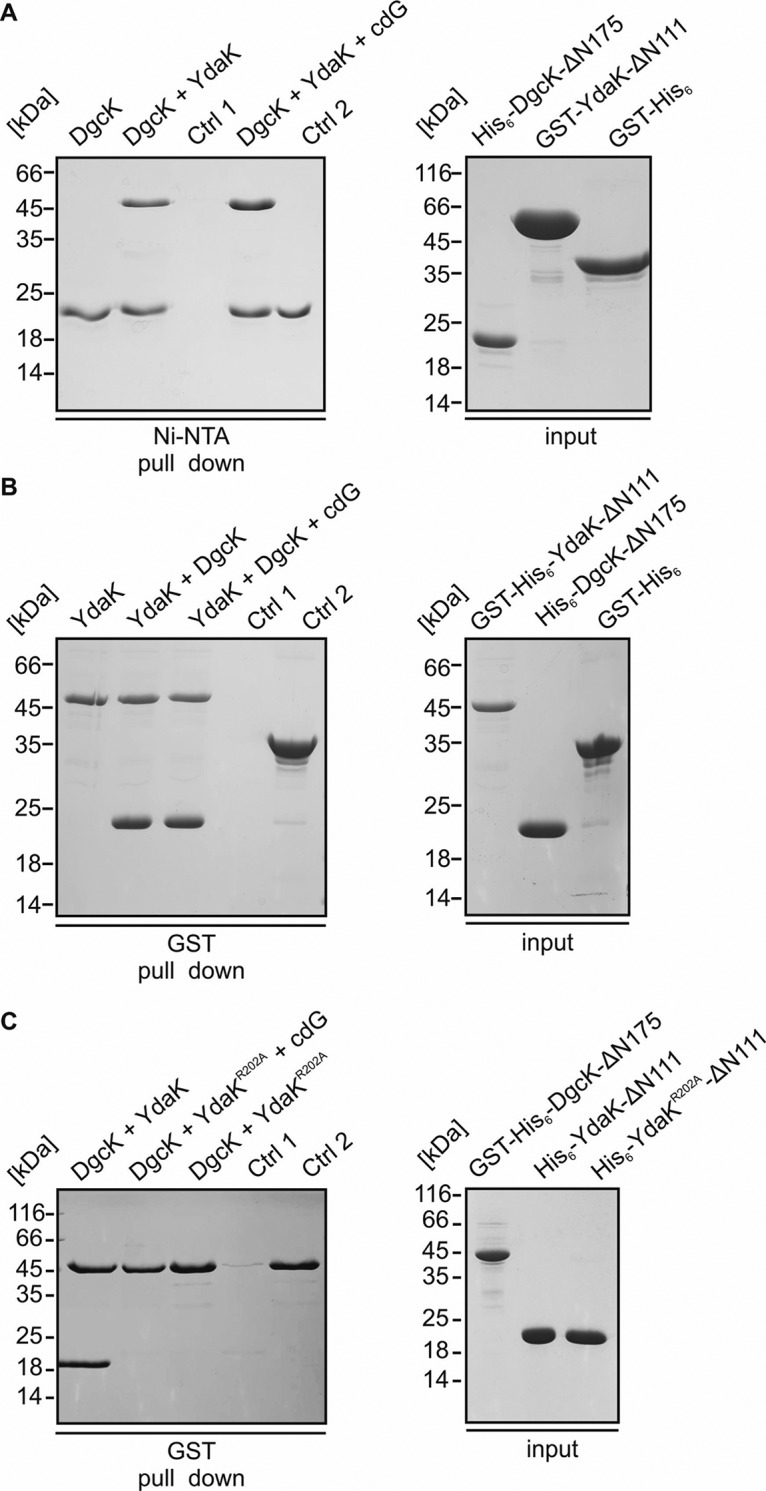
Interaction of the DGC DgcK with its cognate receptor YdaK depends on an intact I-site within the GGDEF domain of YdaK. (A) *In vitro* interaction analysis of His_6_-DgcK-∆N175 and GST-YdaK-∆N111. Coomassie-stained SDS-PAGE of a pull-down assay employing His_6_-DgcK-∆N175 (bait), GST-YdaK-∆N111 (prey) and GST (control 2). 2 nmol His_6_-DgcK-∆N175 was immobilized on nickel-sepharose beads, followed by incubation with 20 nmol GST-YdaK-∆N111 with or without addition of c-di-GMP (cdG, second and fourth lane, respectively). First lane: His_6_-DgcK-∆N175 binding to nickel-sepharose beads. Third lane, Control 1: GST-YdaK-∆N111 does not bind to nickel-sepharose beads. Fifth lane, Control 2: His_6_-DgcK-∆N175 does not bind the affinity tag GST. (B) *In vitro* interaction analysis of GST-His_6_-YdaK-∆N111 and His_6_-DgcK-∆N175. Coomassie-stained SDS-PAGE of a pull-down assay employing GST-His_6_-YdaK-∆N111 (bait), His_6_-DgcK-∆N175 (prey). 2 nmol GST-His_6_-YdaK-∆N111 was immobilized on GST-sepharose beads, followed by incubation with 20 nmol His_6_-DgcK-∆N175 with or without addition of c-di-GMP (cdG, second and third lanes, respectively). First lane: GST-His_6_-YdaK-∆N111 binding to GST beads. Fourth lane: Control 1, His_6_-DgcK-∆N175 does not bind to GST-sepharose beads. Fifth lane: Control 2, His_6_-DgcK-∆N175 does not bind the affinity tag GST. (C) Coomassie-stained SDS-PAGE of a pull-down assay employing GST-His_6_-DgcK-∆N175 (bait), His_6_-YdaK-∆N111 (prey) and the I-site mutant His_6_-YdaK-∆N111_R202A. 2 nmol GST-His_6_-DgcK-∆N175 was immobilized on GST-sepharose beads, followed by incubation with 20 nmol His_6_-YdaK-∆N111 (first lane). Second and third lanes: GST-His_6_-DgcK-∆N175 does not bind His_6_-YdaK-∆N111_R202A with or without additional addition of c-di-GMP (cdG). Fourth lane: Control 1, His_6_-YdaK-∆N111_R202A does not bind to GST-sepharose beads. Fifth lane: Control 2, GST-His_6_-DgcK-∆N175 binding to GST beads.

To further substantiate the idea of a local c-di-GMP pool mediated by direct interaction of DgcK and of YdaK, we purified the corresponding soluble domains of both proteins as hexa-histidine- and glutathione *S*-transferase (GST)-tagged proteins (His_6_-DgcK-ΔN175, GST-His_6_-DgcK-ΔN175, GST-YdaK-ΔN111, GST-His_6_-YdaK-ΔN111). In a first approach (Fig. 4A), immobilized His_6_-DgcK-ΔN175 (on Ni-nitrilotriacetic acid [NTA]-Sepharose) beads retained GST-YdaK-ΔN111 but not purified GST alone, showing that a direct interaction exists between the soluble domains of DgcK and YdaK. To validate these findings further, we performed an inverse-pulldown assay (Fig. 4B) employing immobilized GST-His_6_-YdaK-ΔN111 (on GST-Sepharose beads), which was able to retain His_6_-DgcK-ΔN175 but not the control protein, GST. In a third approach (Fig. S3), His_6_-DgcK-ΔN175 was coexpressed with an untagged version of YdaK-ΔN111 and isolated from E. coli BL21(DE3) cell lysates via its His_6_ tag. MS analysis of the corresponding elution fraction revealed the presence of both components. All three experiments indicate a direct interaction of DgcK-ΔN175 and YdaK-ΔN111 *in vitro*.

10.1128/mBio.03122-19.3 FIG S3 Interaction of DGC DgcK with its cognate receptor YdaK. Coomassie blue-stained SDS-PAGE gel of His_6_-DgcK-ΔN175 and YdaK-ΔN111 coexpressed in E. coli BL21, after Ni-NTA chromatography (diluted elution fraction). Download FIG S3, JPG file, 0.1 MB. Copyright © 2020 Kunz et al. 2020 Kunz et al. This content is distributed under the terms of the Creative Commons Attribution 4.0 International license.

Addition of c-di-GMP in our pulldown experiments had only a minor impact on the interaction of DgcK and YdaK (Fig. 4A and B; see above). However, when we probed our preparations of DgcK and YdaK for the presence of c-di-GMP, we found the nucleotide in large amounts in both DgcK fusion proteins (i.e., His_6_-DgcK-ΔN175 and GST-His_6_-DgcK-ΔN175) but not in YdaK (Fig. S4). To circumvent this and again probe whether the YdaK-DgcK interaction depends on c-di-GMP *in vitro*, we chose a mutational approach. YdaK harbors a conserved I-site (besides a degenerated GGDEF motif) that is known to mediate allosteric product inhibition of active GGDEF domain proteins (18); furthermore, it represents a necessary element within DGC-effector protein interaction areas (6), serving as essential c-di-GMP binding sites for effector proteins, such as degenerated GGDEF domains (19). Indeed, disruption of the I-site of YdaK disrupts signaling between DgcK and YdaK *in vivo* (9). We hypothesized that the I-site motif might determine YdaK’s ability to bind a DgcK fusion variant *in vitro*. Our results indicate that mutation of arginine at position 202 to alanine in YdaK (YdaK_R202A) disrupts the interaction with its cognate DGC, DgcK, as DgcK no longer showed binding to YdaK_R202A, irrespective of the presence of c-di-GMP (Fig. 4C).

10.1128/mBio.03122-19.4 FIG S4 c-di-GMP content of purified proteins. (A and B) DgcK-ΔN175 (A) and YdaK-ΔN111 (B) proteins were denatured with chloroform, and the c-di-GMP was quantified by high-performance liquid chromatography at a wavelength of 260 nm. A solution containing 200 μM c-di-GMP served as the standard. The amount of c-di-GMP quantified equals approximately a loading of 25% of the DgcK molecules. Download FIG S4, TIF file, 0.5 MB. Copyright © 2020 Kunz et al. 2020 Kunz et al. This content is distributed under the terms of the Creative Commons Attribution 4.0 International license.

To further understand how binding of c-di-GMP to the I-site of YdaK might mediate the interaction with DgcK, we performed hydrogen-deuterium exchange mass spectrometry (HDX-MS; explained in the supplemental material, Methods section) with YdaK in the absence and presence of c-di-GMP. Hereby, upon incubation of YdaK in deuterated buffer, the amide protons (H) of the peptide backbone are exchanged for deuterium (D). The amount of deuterium incorporation into the protein is determined after proteolytic digestion of a multitude of peptides, thereby also providing spatial information. The rate of this H/D exchange is influenced by the “neighborhood” of each amide proton (i.e., mainly their engagement in hydrogen-bonding interactions), and thus, alterations in H/D exchange are indicative of different conformational states of the protein.

**FIG 5:**
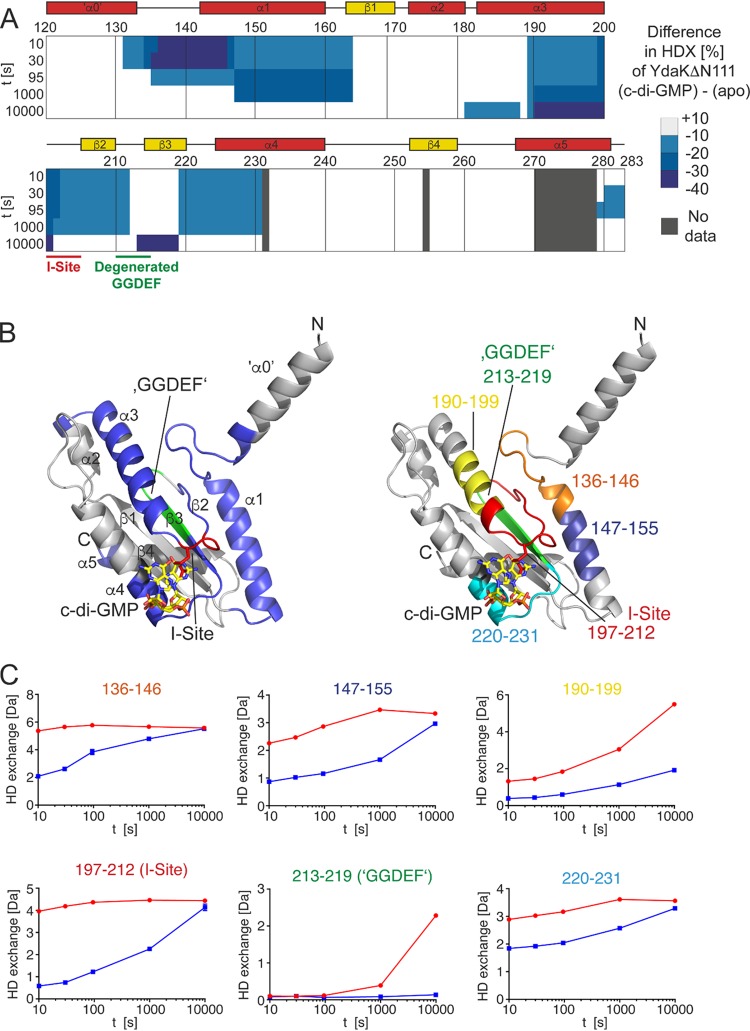
c-di-GMP alters the conformation of YdaK. (A) Amino acid residues of YdaK-ΔN111 are colored according to their differences in HDX profiles between c-di-GMP-bound YdaK-ΔN111 and apo-YdaK-ΔN111. The secondary structure of YdaK-ΔN111 based on a generated model is indicated. (B, left) Locations of regions with less HDX in the presence of c-di-GMP in a structural model of YdaK-ΔN111. The I-site and degenerated GGDEF motifs are in red and green, respectively. The I-site arginine 202 is shown as sticks. The position of c-di-GMP bound to the I-site is inferred from a superimposition of the YdaK-ΔN111 structural model upon the crystal structure of the GGDEF domain of Dcsbis from Pseudomonas aeruginosa (PDB accession number 4ZMM). (Right) Location of representative peptides in the structural model of YdaK-ΔN111. (C) Hydrogen/deuterium exchange profiles of representative YdaK peptides in the c-di-GMP-bound (red) and unliganded (blue) states. Data represent the means ± standard deviations (SD) of results from three technical replicates. t, time.

In the HDX-MS experiment of native YdaK, we could retrieve a total of 61 peptides that cover 88.3% of the amino acid sequence of YdaK, with approximately 5-fold redundancy per amino acid (see Dataset S1 in the supplemental material). The presence of c-di-GMP resulted in a strong decrease in H/D exchange in many parts of YdaK compared to what occurred in the unbound state (Fig. 5A and C). We decided to generate a structural model of YdaK with SWISS-MODEL based on the crystal structure of a GGDEF domain containing bacteriophytochrome (PDB accession number 5LLX) (20) to extend our view. The c-di-GMP-caused conformational changes encompassed (i) the linker between the α-helix preceding the GGDEF domain (α0) and the first helix of the GGDEF domain (α1), (ii) the entire α1 helix, (iii) the entire α3 helix anterior to the I-site, (iv) the I-site motif RXXD, containing the conserved arginine 202 residue, (v) the degenerated GGDEF motif located at the tip of the β1-β2 hairpin, and (vi) the N-terminal part of α3 (Fig. 5A and B). While differences close to the I-site may simply have been caused by coordination of the c-di-GMP, other more-remote regions of difference (i.e., the α0/α1 linker region and the β1-β2 hairpin) (Fig. 5B and C) imply a major conformational change of the whole GGDEF domain following the coordination of c-di-GMP.

10.1128/mBio.03122-19.10 DATASET S1 HDX-MS data from HDX experiments. Download Data Set S1, XLSX file, 0.2 MB. Copyright © 2020 Kunz et al. 2020 Kunz et al. This content is distributed under the terms of the Creative Commons Attribution 4.0 International license.

We finally also probed by HDX-MS whether the R202A variant of YdaK responds to c-di-GMP. In contrast to the drastic changes observed for native YdaK (see above), no alterations in the conformation of YdaK_R202A were observed, indicating the inability of c-di-GMP to bind to this variant (Fig. S5A and B). The loss of interaction with c-di-GMP is not caused by a defective conformation of YdaK_R202A because it exhibits the same H/D exchange dynamics as native YdaK in the absence of c-di-GMP (Fig. S5C and D). Taken together, our experiments evidence that coordination of c-di-GMP at the I-site of YdaK is a prerequisite for its interaction with DgcK.

10.1128/mBio.03122-19.5 FIG S5 YdaK_R202A is not responsive to c-di-GMP. (A and B) Difference in the hydrogen/deuterium exchange profiles of wild-type YdaK-ΔN111 (A) and YdaK-ΔN111_R202A (B) in the presence and absence of c-di-GMP. (C and D) Native YdaK and its R202A variant harbor similar conformations. (C) Relative hydrogen/deuterium exchange profiles of YdaK-ΔN111_R202A (top) and wild-type YdaK-ΔN111 (bottom) in the absence of c-di-GMP; (D) difference in the hydrogen/deuterium exchange profiles of YdaK-ΔN111_R202A and wild-type YdaK-ΔN111 in the absence of c-di-GMP. Download FIG S5, TIF file, 1.5 MB. Copyright © 2020 Kunz et al. 2020 Kunz et al. This content is distributed under the terms of the Creative Commons Attribution 4.0 International license.

### All three Dgc proteins show individual contributions toward inhibition of motility.

Swarming motility is inhibited by DgrA in response to elevated intracellular c-di-GMP levels, as a consequence of a *pdeH* deletion or of an overexpression of DgcK, DgcP, and DgcW-ΔEAL. The transient reduction of motility in *pdeH* mutants could be rescued by an additional mutation in *dgrA* or by the simultaneous disruption of all three DGCs (5, 12), but it is still unclear which particular DGC contributes to the inhibition of motility via DgrA.

**FIG 6:**
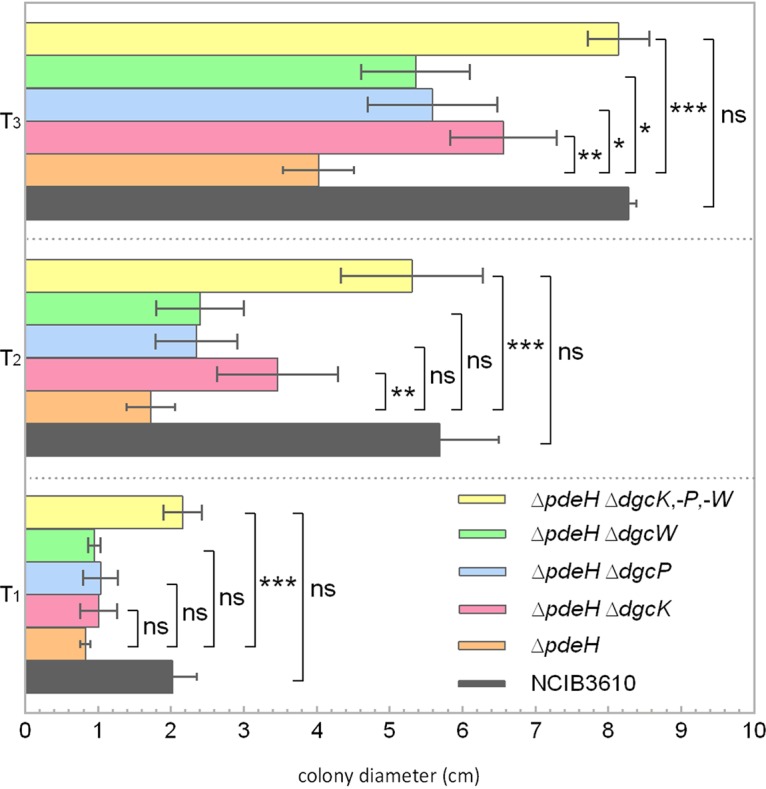
Swarm expansion assays of *pdeH* mutant strains harboring additional *dgc* gene deletions in B. subtilis NCIB 3610. The defect in swarming motility of the *pdeH* mutant can be rescued by deleting all three *dgc* genes. Semiquantitative colony expansion assay of wild-type B. subtilis NCIB 3610 and of strains with *pdeH* deleted combined with additional deletions of the indicated *dgc* genes (DK391, *pdeH*; DS9305-DK391, Δ*dgcK* Δ*pdeH*; DS9537-DK391, Δ*dgcP* Δ*pdeH*; DS9883-DK391, Δ*dgcW* Δ*pdeH*; DS1809-DK391, Δ*dgcK* Δ*dgcP* Δ*dgcW* Δ*pdeH*) on 0.7% (wt/vol) LB agar for 1 h (T_1_), 2 h (T_2_), and 3 h (T_3_) after incubation at 37°C. All values are the averages of results from at least four experiments, which included three biological replicates. The colony diameters of all strains ranged between 0.8 and 0.9 cm at time zero. Asterisks indicate significant differences (*, *P* < 0.05; **, *P* < 0.01; ***, *P* <0.001, two-sided independent *t* test; ns, differences were not significant).

To answer this question, we deleted *pdeH* in all three *dgc* single mutants and in the *dgc* triple mutant. In good agreement with the mentioned studies (5, 12), the *pdeH*-deficient strain showed a clear defect in motility, revealing approximately 60%, 67%, and 50% reductions in the diameter of the colony compared to that of the wild type at 1 h, 2 h, and 3 h postinoculation, respectively (Fig. 6). As expected, the motility defect of the *pdeH* mutant could be rescued by deleting all genes encoding active DGCs (Fig. 6). These results are consistent with the previous observations that wild-type motility can be restored to cells lacking *pdeH* when either DgrA or c-di-GMP production is abolished.

None of the *dgc* single mutants harboring a *pdeH* deletion was able to restore swarming motility after 1, 2, or 3 h of incubation, but all double mutants showed a slight increase in colony diameter size compared to that of the *pdeH* single mutant at time points at 2 h (*T*_2_) and *T*_3_ (Fig. 6). This observation indicates that all three DGCs cooperate in inhibiting motility. All three DGCs are predicted to harbor N-terminal sensor domains, suggesting that integration of different environmental/cellular signals might be achieved.

Although the deletion of single *dgc* genes in a *pdeH* mutant did not result in wild-type motility behavior, deletion of *dgcK* caused more-pronounced effects than other *dgc* gene mutations because the Δ*dgcK* Δ*pdeH* strains swarmed slightly faster than the Δ*dgcP* Δ*pdeH* strain or Δ*dgcW* Δ*pdeH* strain. These results show that DgcK not only plays an important role in EPS production via YadKLMN (9) but also is involved in regulating swarming motility. Swarming inhibition and EPS production seem a plausible outcome of a signal that leads to the cell’s decision to switch to a sessile life style.

### Diffusion of DgcK is increased in the absence of DgrA, based on a direct interaction *in vitro*.

As DgcK mobility is affected by the absence of its receptor YdaK, we asked whether such a direct or close interaction may also exist for DgcK and DgrA and therefore also tracked DgcK-mVenus in a *dgrA* deletion strain. Of note, in Gaussian mixture model (GMM) analyses, diffusion coefficients are adapted to the pair of strains/conditions that are being compared, so the diffusion coefficient of DgcK differs from those determined from the above-mentioned tracking experiments involving YdaK. Strikingly, the mobility of DgcK was strongly increased in the *dgrA* null strain compared to that in wild-type cells (Fig. 3C and E; Table 1), changing from 70% ± 10% static molecules to only 18% ± 2% static molecules, with a diffusion coefficient of 0.075 ± 0.004 μm^2^/s, indicating that in the absence of DgrA, 52% of the DgcK molecules presumably no longer interact with DgrA. On the other hand, the mobile fraction increases from 30% ± 10% static molecules to almost 82% ± 2% static molecules, with a diffusion coefficient of 0.383 ± 0.040 μm^2^/s. Average dwell times of static DgcK molecules decreased from 70 ± 2 ms to 35 ± 2 ms in the absence of PilZ protein DgrA (Fig. 2H and Table 1), which supports our findings that the mobility of DgcK increases in the absence of DgrA and therefore stops less often at the membrane. These data suggest that DgcK also directly or indirectly interacts with the c-di-GMP receptor DgrA.

**FIG 7:**
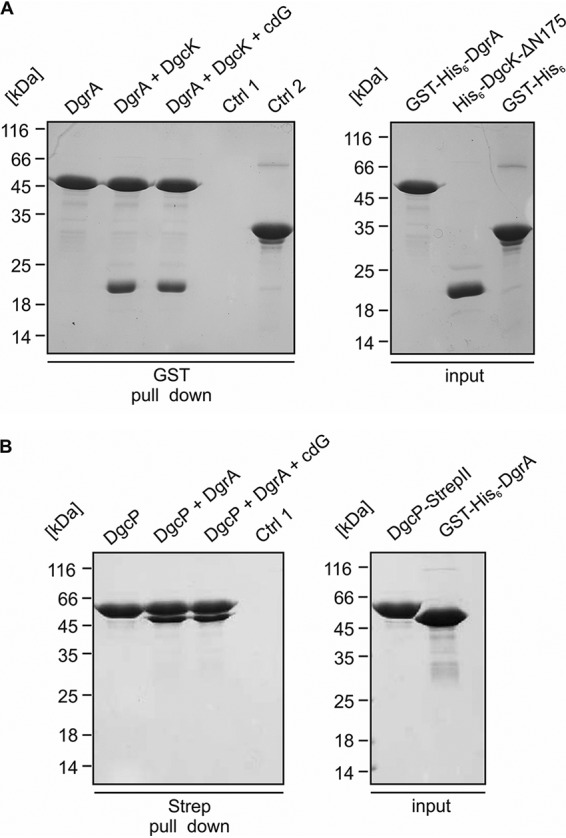
Interaction of the PilZ protein DgrA with DgcK and DgcP. (A, left) *In vitro* interaction analysis of GST-His_6_-DgrA and His_6_-DgcK-ΔN175. Coomassie blue-stained SDS-PAGE gel from a pulldown assay employing GST-His_6_-DgrA (bait), His_6_-DgcK-ΔN175 (prey), and GST (control [Ctrl]). Two nmol of GST-His_6_-DgrA was immobilized on glutathione-Sepharose beads (first lane), followed by incubation with 20 nmol His_6_-DgcK-ΔN175 (second lane) and 2 mM c-di-GMP (cdG) (third lane). Control 1 (fourth lane) is His_6_-DgcK-ΔN175, which does not bind to glutathione-Sepharose beads. Control 2 (fifth lane) is His_6_-DgcK-ΔN175, which does not bind the affinity tag GST. (A, right) Input controls for: GST-His-DgrA (first lane), His-DgcK-ΔN175 (second lane), GST-His_6_ (third lane). (B, left) *In vitro* interaction analysis of DgcP-Strep and GST-His_6_-DgrA. Coomassie blue-stained SDS-PAGE gel from a pulldown assay employing DgcP-StrepII (bait) and GST-His_6_-DgrA (prey). Two nmol of DgcP-StrepII was immobilized on Strep-Sepharose beads (first lane), followed by incubation with 20 nmol GST-His_6_-DgrA (second lane), which shows that DgcP-StrepII can immobilize GST-His_6_-DgrA on Strep-Sepharose. In the third lane, 2 nmol DgcP-Strep was immobilized on Strep-Sepharose beads, followed by incubation with 20 nmol GST-His_6_-DgrA and of 2 mM c-di-GMP. Control 1 (fourth lane) was GST-His6-DgrA, which does not bind to Strep-Sepharose beads. (B, right) Input controls for DgcP-StrepII (first lane), GST-His_6_-DgrA (second lane).

To substantiate this point, we purified DgrA as a GST fusion protein by a two-step protocol and performed affinity pulldown assays. These data show that GST-His_6_-DgrA and His_6_-DgcK-ΔN175 interact *in vitro* (Fig. 7A). We found that His_6_-DgcK-ΔN175 cannot bind to the beads alone (Fig. 7A, lane 4); furthermore, the possibility that His_6_-DgcK-ΔN175 interacts with the GST tag can be excluded (Fig. 7A, lane 5). In summary, single molecule tracking (SMT) and pulldown assays reveal that the DGC DgcK directly interacts with the PilZ receptor DgrA and thereby inhibits motility by synthesizing c-di-GMP at its receptor molecule.

### DgrA affects the motility of the soluble DgcP, based on a direct interaction between cyclase and the receptor.

In contrast to DgcK, DgcP is predicted to be a soluble DGC. Indeed, DgcP-mVenus forms dynamic foci at the cell periphery in time-lapse experiments (9). Total internal reflection fluorescence microscopy (TIRFM) experiments verified that DgcP-mVenus foci are membrane proximal (Fig. S6). Because the absence of DgcP also impacts motility, we analyzed whether the absence of DgrA may influence the dynamics of DgcP, analogously to what was observed for DgcK. We therefore tracked single DgcP-mVenus molecules in wild-type cells (Movie S2) and in *dgrA* mutant cells. In support of the idea of frequent stops at the cell membrane, the heat map of DgcP-mVenus in wild-type cells showed membrane-proximal foci (Fig. S1I and K), in agreement with the results of TIRF analyses (Fig. S6). GMM analyses showed that 65% ± 5% of DgcP-mVenus molecules are static or move slowly, with a diffusion coefficient of 0.07 ± 0.0003 μm^2^/s in wild-type cells, and that 35% ± 5% were dynamic, with a diffusion coefficient of 0.57 ± 0.001 μm^2^/s. In the *dgrA* deletion strain, DgcP-mVenus molecules were considerably more dynamic; only 31% ± 6% were static and 69% ± 6% were dynamic within the cytosol (Fig. 3F to H and Table 3). The loss of membrane-associated DgcP-mVenus molecules can be seen from the heat map, where cytosolically located positions are predominantly seen (Fig. S1J and L). Dwell times of DgcP decreased from 48 ± 4 ms to 30 ± 2 ms due to the lack of DgrA (Fig. 2I and Table 3), revealing that as with what was observed for DgcK, DgcP arrests less often at the cell membrane in the absence of the PilZ domain protein DgrA.

**TABLE 3:** Diffusion coefficients and dwell times of DgcP-mVenus^*a*^

Strain with DgcP-mVenus	Static *D* ± SD (μm² sˉ¹)	Mobile *D* ± SD (μm² sˉ¹)	Static fraction ± SD (%)	Mobile fraction ± SD (%)	Dwell time (s)^*b*^
NCIB 3610	0.07 ± 0.0003	0.57 ± 0.001	65.0 ± 4.8	35.0 ± 4.8	0.048 ± 0.004
Δ*dgrA* strain	0.072 ± 0.0003	0.57 ± 0.001	31.3 ± 6.4	68.7 ± 6.4	0.030 ± 0.002

a The errors were calculated from the results of three biological replicates.

b Dwell time, time period in which a track stays within a radius of 230 nm.

10.1128/mBio.03122-19.6 FIG S6 DgcP-mV-YFP visualized by total internal reflection fluorescence microscopy (TIRFM) reveals distinct focal formation. TIRFM of mid-exponential-phase B. subtilis NCIB 3610 cells expressing *dgcP-mV-yfp* from the amylase locus (strain NCIB 3610-PB86, *amyE*::*P_xyl_-dgcP-mV-yfp*) 45 min after induction with 0.01% (wt/vol) xylose. BF, bright field. Bar = 2 μm. Download FIG S6, TIF file, 0.2 MB. Copyright © 2020 Kunz et al. 2020 Kunz et al. This content is distributed under the terms of the Creative Commons Attribution 4.0 International license.

10.1128/mBio.03122-19.9 MOVIE S2 Exponentially growing B. subtilis cells expressing DgcP-mVenus from the original gene locus. Stream acquisition at 15 ms (66 fps) is shown in slow motion at 10 fps. Download Movie S2, AVI file, 2.1 MB. Copyright © 2020 Kunz et al. 2020 Kunz et al. This content is distributed under the terms of the Creative Commons Attribution 4.0 International license.

We purified DgcP as a soluble, full-length protein to test for a direct association of DgrA and the DGC. Figure 7B shows that DgrA coeluted with immobilized DgcP but did not bind to the streptavidin-binding matrix by itself, showing that DgcP and DgrA also interact *in vitro*.

### Low levels of DgcK and of DgcP molecules in individual cells.

**FIG 8:**
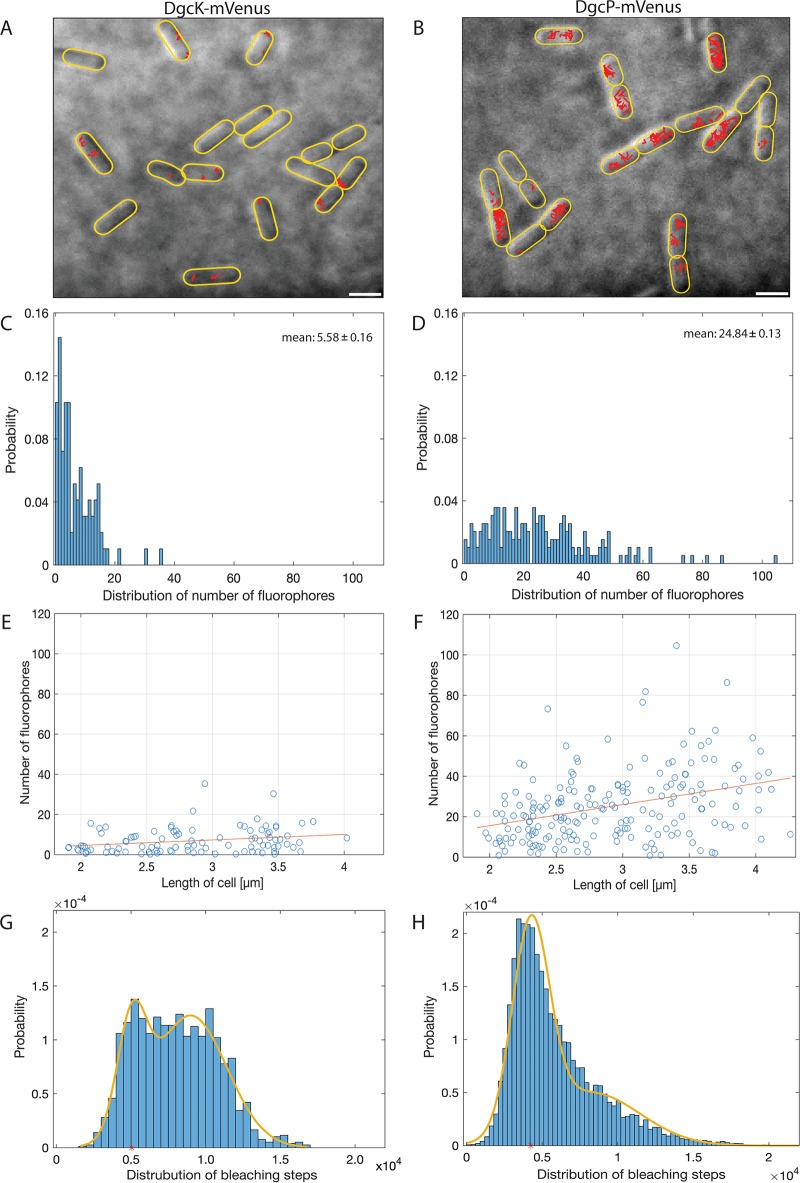
Molecule numbers. (A, B) Projection of tracks in exponentially growing B. subtilis NCIB 3610 wild-type cells onto bright-field images. All tracks of DgcK-mVenus (A) and DgcP-mVenus (B) are shown in red in their respective cells. Scale bars, 2 μm. (C, D) Distribution of the number of fluorophores per cell in exponentially growing cells. Cells expressing DgcK-mVenus (C) harbor on average 5.58 ± 0.16 fluorophores per cell, whereas cells expressing *dgcP-mVenus* (D) show 24.84 ± 0.13 fluorophores per cell. (E, F) Correlation between the number of fluorophores and cell length. For DgcK-mVenus cells (E) there is no correlation between the number of fluorophores and cell size, but a slight correlation can be seen for DgcP-mVenus (F). (G, H) The distributions of bleaching steps for DgcK-mVenus (G) and DgcP-mVenus (H) display two fractions of bleaching steps (monomers and dimers).

During our tracking experiments, we noticed that the number of DgcK-mVenus tracks per cell was very low, much lower than that of other proteins observed in our group (15, 21, 22). Additionally, we noticed that a considerable portion of cells appeared to lack any fluorescence signal (Fig. 8A), indicative of a very low-abundancy protein. To determine the presence or absence of signals corresponding to single molecules and to determine the number of DgcK-mVenus or DgcP-mVenus molecules per cell, we quantified the photon count of single bleaching steps (i.e., single-fluorescent-protein bleaching) toward the end of the acquisitions and divided the total fluorescence intensity at the beginning of the acquisition (not yet bleaching) by that of the single fluorophores, relative to cell size. We developed an algorithm that automatically determines background signals in individual cells and subtracts these from the specific point spread functions from single molecules (for details, see the supplemental material).

Applying this new tool, we verified that 71% of the cells contained DgcK-mVenus fluorescence signals. Thus, a minor but significant fraction of cells lacks DgcK during exponential growth. If DgcK was simply an extremely low-abundancy protein, we would expect a low number of molecules in the remaining cells containing a fluorescent signal, and indeed, in this fraction of cells, the average molecule number was 5.6 ± 0.16 (Fig. 8C). Therefore, due to the low abundance of DgcK, an exponentially growing B. subtilis culture was split into two fractions, DgcK-free cells and cells containing 1 to, rarely, 20 molecules (Fig. 8C). As with DgcK, we determined an average number of 24.8 ± 0.13 molecules/cell for DgcP (Fig. 8D), which was higher but still comparatively low. Both genes are monocistronic and have a predicted sigma A promoter (SubtiWiki). There is no correlation between DgcK molecule number and cell size (Fig. 8E) and only a weak correlation for DgcP (Fig. 8F), suggesting that low and stochastic firing of the promoters leads to cells containing low numbers of molecules to no molecules.

We also determined molecule numbers for YdaK, finding an average low number of 12 molecules per cell (Fig. S7A). As for DgcK, there was no correlation between molecule number and cell size, but the distribution of YdaK numbers was less heterogeneous than for DgcK, as only 7% of cells had a likelihood of containing fewer than 3 YdaK molecules (Fig. S7A), in contrast to 32% of cells with DgcK. Addition of 4% ethanol to cells led to a 20% increase in YdaK molecules (Fig. S7B) and a somewhat more pronounced, but still weak, correlation of molecules and cell length (Fig. S7D). Thus, there are, on average, about or fewer than 2 YdaK receptor molecules per DgcK synthase and about 2.5 molecules after ethanol stress, thus a measurable increase.

10.1128/mBio.03122-19.7 FIG S7 Determination of molecule numbers for YdaK-mVenus. (A and B) Distribution of the numbers of fluorophores per cell in exponentially growing cells. Cells expressing YdaK-mVenus during exponential growth (A) harbor on average 11.8 ± 0.1 fluorophores per cell, whereas cells stressed with 4% ethanol (B) show 14.4 ± 0.9 fluorophores per cell. (C, E) Correlation between the number of fluorophores and cell length during exponential growth (C) or after ethanol stress (D). There is no clear correlation between the number of fluorophores and cell size. Download FIG S7, TIF file, 0.9 MB. Copyright © 2020 Kunz et al. 2020 Kunz et al. This content is distributed under the terms of the Creative Commons Attribution 4.0 International license.

Interestingly, we were able to detect two fractions of molecules with different bleaching steps, one having approximately twice the count of photons as the other (Fig. 8G and H). For DgcK, we observed that 25% of the bleaching molecules have an average bleaching step of 5,047 photons and that 75% of them have 8,975 photons (Fig. 8G). These events likely stem from dimers, where two monomers bleach at the same time and therefore cause the doubling of the number of photons, in agreement with DGCs acting as dimers during c-di-GMP synthesis. The other DGC, DgcP, showed a similar behavior of bleaching events. We found that 60% of the molecules have an average bleaching step of 4,250 photons, whereas the remaining 40% bleach with a roughly doubled number of photons, 8,251 (Fig. 8H). The fact that we observed fewer bleaching events of dimers for DgcP is possibly because of its higher molecule number, which may mask the quantitative detection of individual bleaching steps or because DgcP may diffuse as monomers and form dimers while binding to its receptor (i.e., DgrA).

## DISCUSSION

The multitude of diguanylate cyclases (DGCs) present in many bacterial species (2) and yet the distinct phenotypes observed by disrupting individual cyclase genes have sparked the discussions of how this pathway specificity is achieved using a freely diffusive second messenger. A straightforward explanation would be a close spatial proximity between DGCs, PDEs, and the c-di-GMP receptor molecules, which has been shown for the signaling complex of YdaM (DGC), YciR (PDE), and MlrA (transcription factor) formed in E. coli (7), or even a direct handover (6), generating a local pool, rather than an increase in the overall cellular pool, of c-di-GMP. Our work provides strong support for this idea and shows that c-di-GMP signaling via local pools is a mechanism included in signaling in Bacillus subtilis. We observed increased diffusion of DgcK in the absence of its known receptor, YdaK, and an opposite impact on the diffusion of DgcK upon overexpression of YdaK, which acts on a statically positioned macromolecular nanomachine (8). YdaK is essential for the activity of the *yda* operon, which is assumed to produce an unknown EPS (8). Purified soluble domains of DgcK, an integral membrane protein with 6 transmembrane helices (TMs), and of YdaK, which harbors 4 TMs, interacted with each other as long as the I-site of YdaK was intact, supporting the idea of a direct c-di-GMP-dependent interaction.

Indeed, HDX analyses emphasized the importance of an intact I-site for the interaction; binding of c-di-GMP to YdaK leads to major conformational changes in the entire GGDEF domain and not only at the I-site, which was abolished in the I-site mutant protein. It is also interesting to note that we found purified DgcK to be largely associated with c-di-GMP. Therefore, DgcK may obtain its signal throughout the cell membrane, synthesize c-di-GMP, and diffuse to its receptor, which then undergoes profound conformational changes that may well lead to the activation of the putative EPS nanomachine.

We favor the idea of a local pool not only for signaling toward EPS production but also for the regulation of swarming motility. Our experiments show that DgcK plays a dual role in regulating putative EPS synthesis and inhibition of swarming motility in B. subtilis. This fits with the view that cells would need to reduce motility when they produce an extracellular matrix in a biofilm or during cell aggregation in solution. We found that DgcK becomes more mobile in the absence of the PilZ domain protein DgrA, which acts as a clutch to disengage flagellar rotation (13). DgrA binds to MotA in response to c-di-GMP binding and thereby separates MotA from FliG. This leads to a loss of contact between the flagellar stators and the rotor, so that no torque can be generated. Because purified DgrA showed a direct interaction with the soluble domain of DgcK, our data provide strong support for the concept that DgcK binds to the statically positioned flagellar system to interact with DgrA, which in turn becomes active. Thus, YdaK and DgrA present two diverging branches of signaling via DgcK, involved in motile-to-sessile transformation in order to build biofilms.

Extending our analyses to the soluble DGC DgcP, which also affects swarming motility, we found a strong effect of the absence of DgrA on DgcP single-cell dynamics, in that DgcP stopped much less frequently at the cell membrane in cells devoid of DgrA. Indeed, DgrA might accept c-di-GMP directly from DgcP, based on our finding that the two proteins show a direct interaction *in vitro*. Thus, DgrA receives signal input from two different DGCs, representing convergence of c-di-GMP signaling, which would lead to signal integration.

We point out that while input into signaling via DgrA occurs in a direct manner, at least in the cases of DgcK and DgcP, downregulation occurs via the sole known PDE, PdeH, and thus this aspect of motility may occur via a global pool of c-di-GMP. Further input into the modulation of swimming motility also comes from DgcW, whose absence affects swarming in a way similar to that of DgcK and DgcP, although the contribution of DgcK appears to be the greatest. Thus, all three DGCs in B. subtilis signal into the DgrA receptor. It is still unclear whether DgcP and DgcW have other, individual receptor proteins, such as the third c-di-GMP effector, YkuI.

Our work also provides the quantification of dynamics of DGCs at a single-molecule level. We show that 70% of DgcK and 65% of DgcP molecules are in a static/slow mobility mode, likely involved in binding to their receptors (bound to large macromolecular structures), while the remaining population freely moves within the cell membrane and the cytosol, respectively. Diffusion of free DgcK is slow, while DgcP diffusion is much faster, but the protein has to find its membrane-bound receptors by a 3-dimensional (3D) search, rather than a 2D search, as for DgcK. To quantify the molecule number per individual cell, which is possible by SMTracker, we devised a method for automated fluorophore counting. As each molecule of the DGCs DgcK and DgcP is fused to one mVenus molecule at the original locus, the number of fluorophores corresponds to the number of molecules per cell. By determining the number of DGC molecules per cell, we found low to extremely low copy numbers. The cyclase DgcK was present, with an average of only about 6 molecules per cell, and DgcP was present at 25 molecules per cell. The low molecule number of DgcK resulted in 29% of all cells not containing a single molecule, compared to what occurred with DgcP: in 15% of the cells, no signal could be seen. YdaK was found to be present at about 12 molecules per cell on average but was absent only in a negligible number of cells. The low number of DgcK molecules will lead to heterogeneity in the activity of putative EPS production through the *yda* operon, because DgcK is the major c-di-GMP-based inducer of the activity (9). The low number of DgcK molecules in combination with its colocalization with the slightly higher number of YdaK receptors and with the putative EPS synthetases YdaN and YdaM, mostly at a single site within the cell membrane (8), reveals that a single subcellular site in the cell is employed for signal transduction (possibly including signal perception), regulating enzyme activity (EPS production). This way, cells can take advantage of spatial signal specificity. However, if DgcK were the only DGC talking to DgrA, downregulation of motility would not be efficient, given that the few DgcK molecules (about 6 molecules) would not be able to interact with DgrA with the approximately 26 flagella present in B. subtilis (23), given that 52% are generally engaged in a complex with the receptor DgrA, 13% are engaged with YdaK, and only 30% diffuse to find a new interaction partner. Combined with molecule numbers, it is clear that only 3 molecules of DgcK are in a diffusive mode and that 6 molecules are receptor bound, on average, taking into account cells that contain DgcK. It appears, therefore, that DgcP and DgcW help in this aspect, because all three DGCs cooperate to activate DgrA via c-di-GMP. In fact, this is the case for DgcP, since based on the number of static molecules, 34% of these DGC molecules interact with DgrA. So, convergence of c-di-GMP signal modules appears to be essential to regulate all flagellar systems for adaptation toward biofilm formation. It will be interesting to analyze the dynamics of the DGC DgcW in the presence and absence of DgrA to investigate their impact on swarming motility.

*In toto*, our experiments show how direct DGC/receptor modules can achieve convergent as well as divergent signal transduction in B. subtilis via c-di-GMP and that paired with low molecule numbers, high signaling specificity is achieved.

## MATERIALS AND METHODS

### Bacterial strains and growth conditions.

The nondomesticated Bacillus subtilis strain NCIB 3610 carrying a plasmid-transformable *comI* mutation (24) was used in this study. For cloning, the E. coli strain DH5α was taken. E. coli strains were cultivated at 37°C and 200 rpm in Luria-Bertani (LB) medium, whereas B. subtilis strains were cultivated at 30°C and 200 rpm in LB medium overnight. For single-molecule tracking, cells were incubated at the same temperature and speed in S7_50_ minimal medium (1% [wt/vol] fructose, 0.1% [wt/vol] glutamate, 0.004% [wt/vol] Casamino Acids) (25). To determine the growth rate, the optical density at 600 nm (OD_600_) was measured. For selection, the antibiotics ampicillin (100 μg/ml) and chloramphenicol (5 μg/ml) were added to the culture. To induce the xylose promoter, 0.1% (wt/vol) xylose was added to the cells.

### Strain construction.

B. subtilis strains expressing fluorescent protein (FP) fusions were taken from reverence 9, with yellow fluorescent protein (YFP) being exchanged for mVenus (Tables 4 to 6). In brief, all FP fusions were expressed as C-terminal fusions from the original gene locus, i.e., as the sole source of the protein in the cell and under the control of the original promoter. For strains used for protein purification, see below.

**TABLE 4:** Strains used in this study

Strain	Relevant genotype	Source, reference, or description
DK1042 (NCIB 3610)	Prototroph, *comI*^Q12L^	Gift from D. Kearns

Derivatives of NCIB 3610 (DK1042, transformed with plasmid DNA)		
NCIB 3610-PB01	P*_dgcK_*-*dgcK*-*mV*-*yfp* (*cat*) *comI*^Q12L^	This study
NCIB 3610-PB08	P*_dgcP_*-*dgcP*-*mV*-*yfp* (*cat*) *comI*^Q12L^	This study
NCIB 3610-SK01	P*_xyl_*-*ydaKLMN* (*spec*) *comI*^Q12L^	This study

Derivatives of NCIB 3610 (DK1042, transformed with chromosomal DNA from DS9289 and plasmid DNA)		
DS9289	Δ*ydaK*	12
DS9289-PB01	Δ*ydaK* P*_dgcK_*-*dgcK*-*mV*-*yfp* (*cat*) *comI*^Q12L^	PB01→(DS9289→DK1042)
DS9289-PB08	Δ*ydaK* P*_dgcP_*-*dgcP*-*mV*-*yfp* (*cat*) *comI*^Q12L^	PB08→(DS9289→DK1042)
BKK22910	Δ*dgrA*	BGSC
BKK22910-PB01	Δ*dgrA* P*_dgcK_*-*dgcK*-*mV*-*yfp* (*cat*) *comI*^Q12L^	PB01→(BKK22910→DK1042)
BKK22910-PB08	Δ*dgrA* P*_dgcP_*-*dgcP*-*mV*-*yfp* (*cat*) *comI*^Q12L^	PB08→(BKK22910→DK1042)

**TABLE 5:** B. subtilis plasmids and vectors used in this study

Plasmid or vector	Relevant genotype	Source
pSG1164	*bla cat yfp*	43
pSG1164-PB01	P*_dgcK_*-*dgcK*-*mV*-*yfp* (*cat*)	9
pSG1164-PB08	P*_dgcP_*-*dgcP*-*mV*-*yfp* (*cat*)	9
pSG1164-SK01-pCM::sp	P*_xyl_*-*ydaKLMN* (*spec*)	This study

**TABLE 6:** E. coli plasmids and vectors used in this study

Vector	Relevant fusion	Plasmid^*a*^
pGAT3	GST-His_6_-DgcK-ΔN175	pAT02a
pET24d	His_6_-DgcK-ΔN175	pAT02b
pGAT3	GST-His_6_-YdaK-ΔN111	pAT03a
pET24d	His_6_-YdaK-ΔN111	pAT03b
pGAT3	GST-YdaK-ΔN111	pAT03c
pET16d	YdaK-ΔN111	pAT03h
pPR-IBA101	DgcP-StrepII	pPB25
pGAT3	GST-His_6_-DgrA	pPB48

a All plasmids were from this study.

### Single-molecule tracking, data acquisition, and analyses.

Cells were grown in S7_50_ minimal medium under selective pressure to the exponential growth phase (OD_600_ between 0.5 and 0.6) at 30°C. To induce the transcription of the *ydaKLMN* operon, cells (NCIB 3610 P*_xyl_*-*ydaKLMN dgcK-mVenus*) were stressed with 4% ethanol for 30 min before microscopy. Afterwards, 5 μl of cells was place onto a coverslip and fixed by a thin agarose pad (1% [wt/vol] agarose in S7_50_ minimal medium). The images were taken with a Nikon Eclipse Ti microscope equipped with a high-numerical-aperture (NA) objective (CFI Apochromat TIRF 100× oil, NA = 1.49), an electron microscope charge-coupled device (EM-CCD) camera (Hamamatsu ImagEMX2), and a YFP filter set (BrightLine 500/24, Beamsplitter 520, and BrightLine 542/27). YFP fluorophores were excited by the central part of a laser beam (TOPTICA Beam Smart, 515 nm; maximum power, 100 mW) with a laser intensity of 20 mW. Each movie consists of 3,000 frames and was recorded at a frame rate of approximately 65 Hz using VisiView (Visitron Systems).

Movies were cut in Fiji (ImageJ) (26) to equal frame lengths of approximately 2,000 frames. Afterwards, the cell meshes were set in Oufti (27). For the following particle tracking, the MATLAB software u-Track (28) was used. The minimal length of the tracks was set to 4, and no gaps in the particle trajectories between two linked points were allowed. The single-molecule tracking data were analyzed and visualized with the MATLAB software SMTracker (29) using the import/exploratory panel, the Gaussian mixture model (GMM) analysis panel, and the spatial distribution assay (SDA) panel. The percentage of cells with no detected tracks was determined using the display movie data import/exploratory panel. The GMM tab was used for subpopulation analysis. The diffusion coefficients and the fraction sizes of the subpopulations were calculated on the assumption that Brownian motion is a Gaussian process with a normal distribution. The heat maps of trajectory location without mirroring axes were taken from the SDA panel.

### Quantification of the number of fluorophores per cell.

The number of fluorophores per cell was determined using a tool of the MATLAB software SMTracker (29). First, the integrated intensity of the cell directly after the initial laser illumination was measured. Therefore, the average of the integrated intensity of the first two frames was taken and subtracted by the background (30) as well as corrected by the uneven illumination and the autofluorescence of the cell. The obtained value was afterwards divided by the fluorescence intensity of the bleaching step of a single fluorophore (31, 32). This new tool was integrated into the SMTracker program.

### Swarming assay.

Swarming motility assays of different B. subtilis mutants were performed using soft agar plates (33). The soft agar plates, which are prepared 1 day before use, consisted of 25 ml LB medium and 0.7% (wt/vol) Bacto agar. Prior to inoculation, the swarm plates were dried for 20 min in a laminar flow hood to minimize the water content and for 15 min postinoculation. One milliliter of cells grown to mid-exponential phase (OD_600_ between 0.6 and 0.8) was resuspended in PBS buffer (8 g/liter NaCl, 0.2 g/liter KCl, 1.44 g/liter Na_2_HPO_4_, 0.24 g/liter KH_2_PO_4_, pH 7.0) to an OD_600_ of 10 and spotted on the center of a soft agar plate. To ensure a constant humidity during swarming, the swarm plate was placed into a lager petri dish with a 14-cm diameter containing 5 ml of water and afterwards incubated at 37°C. The diameters of the colonies were measured hourly.

### Pulldown experiments.

Protein purification is described in the supplemental material. GST pulldown assays were performed at 4°C in buffer A containing 20 mM HEPES-Na (pH 7.5), 250 mM NaCl, 20 mM MgCl_2_, 20 mM KCl, and 0.05% Tween. Purified GST-protein (2 nmol) was applied to 15 μl glutathione-Sepharose 4B (GE Healthcare) in small filter columns (MoBiTec) by incubation on a wheel for 15 min. Subsequently, 20 nmol of the putative binding partner and 2.5 mM c-di-GMP were added and incubated for 10 min at 4°C on a turning wheel. After centrifugation (3,500 × *g*, for 1 min at 4°C) the column was washed three times with buffer A. Proteins were eluted with 40 μl of GSH buffer (50 mM Tris-HCl, 20 mM glutathione [pH 7.5]) and analyzed by Coomassie blue-stained SDS/PAGE.

Ni-NTA and Strep-Tactin affinity binding assays were performed by employing Ni-Sepharose 6 fast-flow beads and Strep-Tactin–Sepharose resin, respectively. His_6_ fusion proteins were eluted using buffer A complemented with 500 mM imidazole, whereas Strep fusions were eluted with buffer A containing 50 mM d-biotin.

### Determination of the c-di-GMP content of protein preparations.

Proteins were denatured by mixing 50 μl of a protein solution (at a typical concentration of approximately 500 μM) with 100 μl chloroform. The mixtures were agitated for 15 s, incubated at 95°C for 15 s, and then flash-frozen in liquid nitrogen. After removal from the liquid nitrogen, the samples were thawed and centrifuged (17,300 × *g*, 30 min, 4°C). The aqueous phases were removed and analyzed by high-performance liquid chromatography on an Agilent 1260 series system (Agilent Technologies) equipped with a C_18_ column (EC 250/4.6 Nucleodur HTec, 3 μM; Macherey-Nagel). Nucleotides were eluted at a 0.8-ml/min flow rate with a buffer containing 50 mM KH_2_PO_4_, 50 mM K_2_HPO_4_, 15 mM tetrabutylammonium bromide, and 15% (vol/vol) acetonitrile and detected at a 260-nm wavelength. The amount of c-di-GMP contained in the protein preparations was quantified using a c-di-GMP standard (Jena Bioscience) with known concentrations as a reference.

### Structural model of YdaK-ΔN111.

A structural model of YdaK-ΔN111 was generated with SWISS-MODEL (34–36) and Swiss-PdbViewer (37, 38). For search of a suitable template, the template library version 2019-10-17, Protein Data Bank (PDB) release of 11 October 2019, was utilized and 324 templates were found. The structural model of YdaK-ΔN111 was generated based on the crystal structure of a GGDEF domain-containing bacteriophytochrome (PDB accession number 5LLX [20]) and had the following quality parameters: GMQE (global model quality estimation) was 0.65, QMEAN (qualitative model energy analysis) was −1.58, Cβ was 0.09, All Atom was 1.19, solvation was 1.20, and torsion was −2.14. SWISS-MODEL proposes a homodimer of YdaK-ΔN111 with a QSQE (quaternary structure quality estimate) score of 0.40.

### HDX-MS.

Hydrogen-deuterium exchange mass spectrometry (HDX-MS) experiments with YdaK-ΔN111 and the YdaK-ΔN111_R202A variant were carried out in the absence or presence of c-di-GMP. The proteins and c-di-GMP were mixed to reach final concentrations of 50 μM and 1 mM, respectively, incubated for 5 min at 25°C and directly used for HDX-MS. Sample preparation was aided by a two-arm robotic autosampler (Leap Technologies). Seven point five microliters (50 μM) of YdaK-ΔN111 or the YdaK-ΔN111_R202A variant with or without c-di-GMP was mixed with 67.5 μl of D_2_O-containing buffer (20 mM HEPES-Na, pH 7.5, 20 mM MgCl_2_, 20 mM KCl, 500 mM NaCl) to start the H/D exchange. After 10-, 30-, 95-, 1,000-, and 10,000-s incubations at 25°C, 55 μl of the reaction mixture was mixed with an equal volume of quench buffer (400 mM KH_2_PO_4_-H_3_PO_4_, 2 M guanidine-HCl, pH 2.2) that had been prechilled at 1°C, and 95 μl of the resulting mixture was immediately injected into an Acquity ultrahigh-performance liquid chromatography (UPLC) M-class system with HDX Technology (Waters). Undeuterated samples were prepared similarly by 10-fold dilution in H_2_O-containing buffer. Proteins were digested on line with immobilized porcine pepsin at 12°C with a constant flow (100 μl/min) of water plus 0.1% (vol/vol) formic acid, and the resulting peptic peptides were collected on a trap column (2 mm by 2 cm) filled with POROS 20 R2 material (Thermo Scientific) kept at 0.5°C. After 3 min, the trap column was placed in line with an Acquity UPLC bridged ethylsiloxane-silica hybrid (BEH) C_18_ column (1.7 μm 1.0 by 100 mm; Waters), and the peptides were eluted at 0.5°C using gradients of water plus 0.1% (vol/vol) formic acid (gradient A) and acetonitrile plus 0.1% (vol/vol) formic acid (gradient B) at a 30-μl/min flow rate as follows: 0 to 7 min with 95 to 65% gradient A, 7 to 8 min with 65 to 15% gradient A, and 8 to 10 min with 15% gradient A. Peptides were ionized by electrospray ionization (capillary temperature, 250°C; spray voltage, 3.0 kV) and mass spectra acquired over a range of 50 to 2,000 *m/z* on a Synapt G2-Si mass spectrometer with ion mobility separation (Waters) in high-definition MS with elevated energy (HDMS^E^) or HDMS mode for undeuterated and deuterated samples, respectively. Lock mass correction was conducted with a [Glu1]-fibrinopeptide B standard (Waters). During separation of the peptides, the pepsin column was washed three times with 80 μl of 4% (vol/vol) acetonitrile and 0.5 M guanidine hydrochloride, and additionally, blank injections were performed between each sample. All measurements were carried out in triplicate.

Peptides were identified with ProteinLynx Global Server 3.0.1 (PLGS; Waters) as described previously (39) from the nondeuterated samples acquired with HDMS^E^ by employing low-energy, elevated-energy, and intensity thresholds of 300, 100, and 1,000 counts, respectively. Peptides were matched using a database containing the amino acid sequences of YdaK-ΔN111 or YdaK-ΔN111_R202A, pepsin, and their reversed sequences. The search parameters were as follows: the peptide tolerance was automatic, the fragment tolerance was automatic, the minimum number of fragment ion matches per peptide was 1, the minimum number of fragment ion matches per protein was 7, the minimum number of peptide matches per protein was 3, the maximum number of hits to return was 20, the maximum protein mass was 250,000, the primary digest reagent was nonspecific, the number of missed cleavages was 0, and the false-discovery rate was 100. For quantification of deuterium incorporation with DynamX 3.0 (Waters), peptides had to fulfil the following criteria: a minimum intensity of 10,000 counts, a maximum length of 30 amino acids, a minimum number of products of 3, a minimum number of products per amino acid of 0.05, a maximum mass error of 25 ppm, and a retention time tolerance of 0.5 min. After automated data processing by DynamX, all spectra were manually inspected and, if necessary, peptides omitted (e.g., in case of a low signal-to-noise ratio or the presence of overlapping peptides). Plots of hydrogen/deuterium exchange profiles were generated with MEMHDX (40).

### Protocol for automated copy number quantification of fluorophores using bleaching curve assays.

In principle, the quantification of fluorophores is based in two basic steps: the estimation of the bleaching step of a single fluorophore (step A) and the measurement of the integrated intensity of the cell right after the laser illuminates the sample (step B). Dividing the latter by the former will give us a good estimation of the number of fluorophores presented inside a cell. A more detailed explanation can be found below.

**(i) Estimation of a single bleaching step.** First, do a regular tracking procedure, selecting the minimum length of the track of 3 frames. For every frame that holds a track, the intensity is calculated as follows:

a. Apply illumination correction and background subtraction to the frame.
b. Set two masks, one circled mask centered on the spot’s exact location, with a radius of 6 pixels, and the other mask with the cell contour extended to 2 pixels. The median of the number of pixels outside the inner circle but inside the cell is considered the whole contribution of background and subtracted from the intensity inside the inner circle.
c. The integrated intensity of the spot is the sum of the intensity per pixel inside the inner circle.
d. Move forward to the next frame and back to step a.
e. Once the last frame of this track is reached, a median filter to the intensity data versus time is applied to clean noisy data. Every resulting intensity is stored.
f. A multiple-Gaussian-peak fit to the histogram of spot intensities is used to infer the intensity of a single fluorophore. The mean of the Gaussian component with the lower-order peak is considered the best estimate for the unitary step size (31, 32), which suggests the use of the gamma mode, but it works only if only one peak is found.

**(ii) Initial integrated intensity.** After background subtraction and correction for uneven illumination and autofluorescence contribution, the average of the values for the first two frames after the laser is on is considered.

**(iii) Uneven-illumination correction (ratio).** In order to correct uneven illumination along the focused field, a ratio matrix was constructed. Using movies without any cells previously corrected by instrumental background, the mean of the intensities for every pixel after the laser was on was calculated and divided by the maximum intensity. This gives for every pixel a ratio with values between 0 and 1. This value must divide every frame.

**(iv) Instrumental background subtraction (offset).** The median of the values for the first 20 frames before the laser is on is subtracted from each frame (30).

**(v) Autofluorescence contribution.** To estimate the amount of fluorescence that comes from cell autofluorescence, a quadratic-regression model of the background signal and autofluorescence signal in wild-type cells using several movies (typically 10) was applied. Then, by implementing the same model for the movies containing cells with tagged proteins, we recovered the autofluorescence contribution at the moment that the laser was switched on. Considering both types of correction, the measured intensity that results is calculated with the following equation: intensity = (observed intensity – offset)/ratio.

### Biofilm formation assay.

Undomesticated B. subtilis NCIB 3610 strains were cultured in LB medium containing appropriate antibiotics at 30°C for 14 h. Daily cultures were grown in LB medium at 37°C to an OD_600_ of 1.0 without antibiotics. For biofilm growth, bacteria from a liquid LB culture were collected and transferred to liquid MSgg medium [5 mM potassium phosphate (pH 7.0), 100 mM 3-(*N*-morpholino)propanesulfonic acid (pH 7.0), 2 mM MgCl_2_, 700 μM CaCl_2_, 50 μM MnCl_2_, 100 μM FeCl_3_, 1 μM ZnCl_2_, 2 μM thiamine, 0.5% glycerol, 0.5% glutamate]. Cells were incubated for an additional 30 min at 37°C and at 200 rpm before inoculation (2 μl) on MSgg plates (MSgg medium fortified with 1.5% Bacto agar on 6-well plates that had been dried overnight) supplemented with Congo red (40 μg/ml) and Coomassie brilliant blue (20 μg/ml) and with 0.1% (wt/vol) xylose and/or 1 mM IPTG (isopropyl-β-D-thiogalactopyranoside) (41, 42). Plates were sealed and incubated for up to 72 h at 25°C. Colony morphology was documented over time using the ChemiDoc MP system (Bio-Rad). For each strain, we analyzed three biological replicates in at least two independent experiments.

### Heterologous protein production and purification.

For recombinant protein overproduction in E. coli BL21(DE3), the commercial expression vectors pET24d (for N-terminal His_6_ fusions), pET16d (for untagged protein variants), pGAT3 (for N-terminal GST fusions), and pPR-IBA101 (for C-terminal StrepII fusions) were used. Coding sequences for all clones were amplified from B. subtilis strain NCIB 3610 genomic DNA using standard PCR techniques. Details of the resultant (fusion) proteins from expression plasmids are the following: GST-His_6_-DgcK-ΔN175 (construct pAT02a, residues 176 to 359), His_6_-DgcK-ΔN175 (construct pAT02b, residues 176 to 359), GST-His_6_-YdaK-ΔN111 (construct pAT03a, residues 112 to 283), His_6_-YdaK-ΔN111 (construct pAT03b, residues 112 to 283), His_6_-YdaK-ΔN111_R202A (construct pAT03b, residues 112 to 283; I-site mutant R202A), GST-YdaK-ΔN111 (construct pAT03c, residues 112 to 283), YdaK-ΔN111 (construct pAT03h, residues 112 to 283), DgcP-StrepII (construct pPB25, residues 1 to 579; full length), and GST-His_6_-DgrA (construct pPB48, residues 1 to 217; full length).

Constructs were transformed in E. coli BL21(DE3) for overexpression, and cells were grown in lysogeny broth medium supplemented with d-(+)-lactose-monohydrate (12.5 g/liter), kanamycin (50 mg/liter), and/or ampicillin (100 mg/liter). After an incubation of approximately 16 h at 30°C under rigorous shaking, cells were harvested by centrifugation (3,500 × *g*, 20 min, 4°C).

All fusion proteins were subjected to a two-step purification procedure, including affinity chromatography and size exclusion chromatography (SEC). Prior to Ni-NTA chromatography, cell pellets for all His_6_-YdaK fusion proteins and its variants were resuspended in equilibration buffer containing 20 mM HEPES (pH 8.0), 500 mM NaCl, 20 mM MgCl_2_, 20 mM KCl, and 40 mM imidazole. Subsequently, cells were lysed using the M-110L Microfluidizer (Microfluidics). After centrifugation (63,000 × *g*, 20 min, 4°C), clarified lysates were applied to a 1-ml HisTrap fast-flow (FF) column (GE Healthcare) equilibrated with 10 column volumes of equilibration buffer. After cells were washed with 60 ml equilibration buffer, proteins were eluted using 15 ml elution buffer (equilibration buffer containing 500 mM imidazole). Elution fractions containing protein were concentrated using Amicon Ultracel-10K (Millipore) and subsequently subjected to SEC (HiLoad 26/600 Superdex, 200 pg; GE Healthcare) with equilibration in SEC buffer (20 mM HEPES [pH 7.5], 500 mM NaCl, 20 mM MgCl_2_, and 20 mM KCl). Fractions were analyzed using SDS-PAGE. Protein-containing fractions were pooled and concentrated. Concentration was determined by a spectrophotometer (NanoDrop Lite; Thermo Scientific).

Similarly, all DgcK and DgrA fusion proteins including an N-terminal His_6_ tag were purified via Ni-NTA chromatography prior to SEC using Tris-based buffers. For DgcK, the following buffers were used: equilibration buffer containing 25 mM Tris-HCl (pH 8.0), 500 mM NaCl, 10% (wt/vol) glycerol, and 40 mM imidazole; elution buffer containing 500 mM imidazole; and SEC buffer containing 50 mM Tris-HCl (pH 8.0) and 250 mM NaCl. The buffer composition for DgrA purification was equilibration buffer (25 mM Tris-HCl [pH 8.0], 300 mM NaCl, 40 mM imidazole), elution buffer (equilibration buffer containing 500 mM imidazole), and SEC buffer (50 mM Tris-HCl [pH 8.0], 250 mM NaCl).

The GST-YdaK-ΔN111 protein was purified using an affinity GSTrap FF column (GE Healthcare) and the following buffers: equilibration buffer, SEC buffer (50 mM Tris-HCl [pH 8.0], 250 mM NaCl), and elution buffer (equilibration buffer containing 20 mM GSH buffer). Prior to SEC (50 mM Tris-HCl [pH 8.0], 150 mM NaCl), DgcP-StrepII fusion proteins were isolated from clear cell lysates employing StrepTrap high-performance (HP) columns.

Truncated variants of DgcK and YdaK were coproduced and copurified via a His_6_ tag at the N-terminus of DgcK. Briefly, gene expression, mediated by two different plasmids was allowed for 16 h at 30°C. After cell disruption, potential protein complexes were enriched via NI-NTA chromatography.
